# Characterization of Long-Term Cultured Murine Submandibular Gland Epithelial Cells

**DOI:** 10.1371/journal.pone.0147407

**Published:** 2016-01-22

**Authors:** Kazuhiro Ikeura, Tetsuya Kawakita, Kazuyuki Tsunoda, Taneaki Nakagawa, Kazuo Tsubota

**Affiliations:** 1 Department of Ophthalmology, Keio University School of Medicine, Tokyo, Japan; 2 Department of Dentistry and Oral Surgery, Keio University School of Medicine, Tokyo, Japan; Sanford Burnham Medical Research Institute, UNITED STATES

## Abstract

**Purpose:**

Human and rat salivary gland cell lines derived from tumors or genetic modification are currently available for research. Here, we attempted to culture and characterize long-term cultured cells spontaneously derived from wild type murine submandibular glands (SGs).

**Methods:**

SGs were removed from 3-week-old C57B/6J female mice and dissociated by collagenase type 1 and hyaluronidase digestion. Isolated SG epithelial cells were cultured in low calcium, serum-free growth media in the presence of cholera toxin (CT) during early passages. Single-cell colonies were isolated by limiting dilution culture after 25 passages. Early- and late-stage cell cultures were characterized for keratin 14, keratin 18, α-smooth muscle actin, and p63 by immunostaining and quantitative real-time PCR analysis.

**Results:**

SG epithelial cells cultured in optimized media maintained their proliferative ability and morphology for over 80 passages. Long-term cultured cells expressed keratin 14, keratin 18, and p63, indicative of an epithelial phenotype.

**Conclusions:**

Epithelial cells originating from wild type murine SGs could be cultured for longer periods of time and remain phenotypically similar to ductal basal epithelium.

## Introduction

Saliva is essential for maintaining oral health, alimentary bolus formation, and protection of the oral mucous membranes. Salivary gland atrophy caused by Sjogren’s syndrome or following radiation therapy for head and neck cancers can result in hyposalivation and xerostomia that can significantly affect the patient’s quality of life. Xerostomia also increases with age and polypharmacy; thus, this condition may be more prevalent than originally expected.[1]

Oral moisturizers, artificial saliva, and muscarinic-3 receptor stimulants are often prescribed to patients with mild-to-moderate xerostomia.[2] However, these treatments have poor efficacy in patients with severe salivary gland atrophy where reduced salivary flow has much more detrimental effects, including erosion of oral mucous membrane, infections, and dysphagia, which can dramatically impair quality of life. Thus, the development of more effective medical treatments is necessary.[2]

Regenerative treatment might be a potential method to restore the secretory function of atrophic salivary glands. In some animal model studies, functional recovery of salivation was observed after stem-like cells were transplanted into the atrophic glandular tissue.[3] For instance, Lombaert et al. reported that the orthotopic transplant of in vitro cultured salispheres restored saliva production to clinically relevant levels.[4] Many recent studies have reported the therapeutic transplant potential of highly proliferative cells that surround the ducts of naïve salivary glands; [4–6] however, a salivary gland-specific stem cell marker is yet to be detected.[7] This approach might be a promising tool to treat patients with severe salivary gland dysfunction; thus, further optimization of the procedures used to isolate, propagate, and differentiate functional salivary cells is necessary.

Until recently, tumor-derived or immortalized cell lines have been widely used in basic and preclinical research of salivary gland physiology, particularly the HSY[8] and HSG[9] cell lines. HSY cells were established from athymic mice xenograft tumors following transplantation with a human parotid gland adenocarcinoma surgical specimen, whereas HSG cells have been derived from an irradiated human submandibular gland (SG) and are classically used as an in vitro model of salivary gland secretion, morphology, and regeneration.[10, 11] Notably, both HSY and HSG cells exhibit morphological features similar to intercalated duct cells, which function as reserve progenitor cells in the salivary gland.[6] However, these lines are pathophysiologically distinct from normal salivary gland tissue.[12] Cells established from spontaneous tumors can be successfully propagated in vitro and are often used in the study of secretion gland disorder [13–15], yet primary cells derived from wild type murine SGs can subcultured only for a few passages because of their limited growth potential. Despite numerous attempts to establish salivary gland cell lines from normal glandular tissue, no normal, immortalized murine cell line has been reported. Here, we characterized salivary gland epithelial cells cultured long-term without any exogenous genetic modification. An earlier report described an immortal integrin α6β1-expressing cell line spontaneously derived from adult rat salivary progenitor cells that can propagate for more than 400 doublings without losing differentiation potential when cultured in low calcium media supplemented with serum, epidermal growth factor, insulin, transferrin, triiodothyronine, hydrocortisone, adenine, and cholera toxin (CT).[16] Thus, we aimed to isolate a normal mouse SG epithelial cell line using a similar culture system with low calcium and CT.

## Materials and Methods

### Animal Experiments

Animal experiments were performed in accordance with the tenets of the Declaration of Helsinki and the Guidelines for Animal Experimentation of the Japanese Association for Laboratory Animal Science. All procedures were approved by the institutional ethics board of the Keio University School of Medicine (Approval No. 09167)

### Tissue preparation and cell cultures

Three-week-old female C57B/6J mice (CLEA Japan, Tokyo, Japan) were euthanized with ketamine (Ketalar; Sankyou Lifetec Co. Ltd., Tokyo, Japan) and xylazine (Celactal; Bayer Medical Co. Ltd., Tokyo, Japan). Excised SGs were transferred to cold phosphate buffered saline (PBS) containing 5% fetal calf serum (FCS) and 1% penicillin-streptomycin. Excess connective tissue was removed as quickly as possible before mincing in Dulbecco’s modified Eagle’s medium (DMEM, Wako Pure Chemical Industries, Ltd., Osaka, Japan) supplemented with 10% FCS. Minced tissue was incubated with soybean trypsin inhibitor (STI; 10 mg/mL), vortexed for ~1 min at minimum speed, and then digested in 5 mL DMEM containing collagenase type-1 (16.3 mg; Wako), hyaluronidase type I-S (5.7 mg; Sigma-Aldrich, St. Louis, MO, USA), 10% FBS, and DNase I (1 U/mL; Sigma-Aldrich) at 37°C for 1 h with vigorous shaking. After digestion, cells were filtered through a 100-μm mesh nylon cell strainer (BD Biosciences, Franklin Lakes, NJ, USA). Cells that passed through the strainer were centrifuged at 240×g at 4°C for 1 min and washed twice in cold PBS. Finally, the cells were digested with 0.05% trypsin-0.02% EDTA (2.5 mL) and DNase I (0.2 U/mL) at 37°C for 20 min with vigorous shaking. Digested cells were pelleted and plated in a 0.1% gelatin-coated 75-cm^2^ culture flask at a density of 2.6 cells/mm^2^ and cultured in epidermal keratinocyte medium (CnT-07; CELLnTEC Advanced Cell Systems, Bern, Switzerland) plus 0.01mg/mL human recombinant epidermal growth factor (EGF), 2.5mg/mL penicillin-streptomycin, and 0.1mg/mL CT (List Biologic Laboratories Inc., Campbell, CA, USA) and incubated at 37°C in 5% CO_2_. Medium changes were initiated after 3 days, and occurred twice a week thereafter. Once cells became semiconfluent, they were collected using TrypLE Express (Gibco), subcultured at a density of 13.3 cells/mm^2^, and monitored for viability by Trypan blue exclusion (Nacalai Tesque, Tokyo Japan). The procedure was repeated for 80 passages (P80).

### Analysis of cell viability

Cells seeded at a density of 13.3 cells/mm^2^ became semiconfluent in about 7 days. Cell morphology was monitored at each passage and imaged with an Observer.D1 microscope (Carl Zeiss Meditec, Jena, Germany). Culture media was supplemented with CT from primary culture until P2, after Hayflick-limit SG epithelial cells were stably proliferating. Population doubling (PD) were calculated as described in the ATCC^®^ Animal Cell Culture Guide (www.atcc.org). PD times were calculated with following equation: PD = 3.32(log UCY—log I) + X, where UCY, I, and X represent the final cell number, the number of seeded cells, and the doubling level of the inoculum used to initiate the subculture being quantitated, respectively.[17]

### Limiting dilution and colony forming assay

Monolayer cell cultures were dissociated with TrypLE Express treatment, diluted to 10 cells/mL, and then 100 μL cell suspension was transferred to each well of a 96-well plates. Medium (50 μL) was added twice a week thereafter. Cell growth was checked every 12 hours using a phase-contrast microscope. Wells containing a single cell were marked and cultured until they became semiconfluent. Viable cells were transferred to 48- or 24-well plates and further subcultured.

Cell viability between P0 and P80 was assessed by colony forming assay. For this, cells were seeded at a density of 0.1 cells/mm^2^ (100 cells/dish) in 1 mL culture medium. After 7 days, dishes were stained for 60 min with 1% Eosin Y Solution (Muto Pure Chemicals Co., Ltd., Tokyo, Japan) and the number and size of colonies were recorded.

To evaluate cellular transformation in vitro, the soft agar colony formation assay was performed in passages 20, 50, and 80. Bottom agar (0.5%, 12mL; Becton, Dickinson and Company) was prepared for each 6 well plate. Cells were plated in the upper layer of 0.33% agar at a density of 1.0 cells/mm^2^ or 5.2 cells/mm^2^. Colony formation was then observed over a 2 week period.

### Histology and immunohistochemistry

The submandibular and sublingual glands are located together in the anterior neck between the submandibular lymph nodes and sternum. The white fat tissue and sublingual glands were removed to expose the SGs and main duct, which was ligated with a 4–0 nylon suture thread to ensure that only SG cells were isolated. The excised glands were embedded in optimal cutting temperature (OCT; Sakura Finetek Japan Co., Ltd.) compound and frozen in liquid nitrogen. Frozen sections were stained with hematoxylin-eosin (H&E), anti-cytokeratin 14 (ab181595, 1:1,000; Abcam, Cambridge, MA, USA), anti-cytokeratin 18 (ab668, 1:100; Abcam), anti-alpha smooth muscle actin (ab5694, 1:250; Abcam), and anti-human p63 (M7317, 1:50; Dako corporation, Denmark) in 1% BSA in PBS overnight at 4°C.

Alternatively, cultured cells were fixed in 4% paraformaldehyde in PBS for 5 min on ice, transferred to room temperature for 15 min, and then permeabilized for 10 min with 0.25% Triton X-100 (Nacalai Tesque). Non-specific antibody binding was blocked by a 2 h incubation in blocking solution (10% normal goat serum:1% BSA in PBS; 1:1) at room temperature. Antibody incubations were performed as described above. Anti-proliferating cell nuclear antigen (PCNA, M0879, 1:200; DAKO) was used to examine the effect of CT on the proliferation of primary and late-passage cells. Samples were then incubated in Alexa Fluor^®^ 555-conjugated rabbit or mouse IgG secondary antibodies (1:100) for 1 h at room temperature. Hoechst 33342 (1:1,000; Dojindo, Japan) was used as a nuclear counterstain. Slides were imaged with an Observer.D1 microscope.

### Real-time PCR analysis

Total RNA extracted from normal SG tissue and P1 and P80 cell cultures was used for cDNA synthesis with ReverTra Ace^®^ qPCR RT Master Mix (Toyobo Co., Ltd., Tokyo, Japan). qRT-PCR analysis for keratin 14, keratin 18, alpha smooth muscle actin, and p63 expression was performed with a GeneAmp^®^ PCR System 9700 (Applied Biosystems) with the following TaqMan^®^ gene expression primers: *Krt14* (Mm00516876_m1), *Krt18* (Mm01601704_g1), alpha smooth muscle actin (*Acta2*; Mm00725412_s1), p63 (*trp63*; Mm00495788_m1). Values were normalized to *Gapdh* expression using the ΔCT method. Each assay was performed six times for each sample.

### Short Tandem Repeat (STR) analysis

PCR amplification was performed on a GeneAmp^®^ PCR System 9700 (Applied Biosystems). The reaction mixture of 50 μL final volume contained 50 ng cDNA, 5 μL of 10 X PCR Buffer for KOD-Plus-Neo (Toyobo Co., Ltd., Tokyo, Japan), 3 μL of 25 mM MgSO_4_ (Toyobo Co., Ltd., Tokyo, Japan), 5 μL of 2 μM dNTPs (Toyobo Co., Ltd., Tokyo, Japan), 1 μL of KOD-Plus-Neo (Toyobo Co., Ltd., Tokyo, Japan), autoclaved distilled water, forward labeled and reverse primers (STR markers: 18–3, 4–2, 6–7, 9–2, 15–3, 6–4, 12–1, 5–5, X-1, D8S1106, D4S2408 and β-actin). [18] PCR conditions for the multiplex assay was as follows: predenature for 2 min at 94°C, denature for 10 sec at 98°C, extension for 30 sec at 68°C and a final soak at 4°C. PCR products were screened by using gel electrophoresis. 10 X Loading Buffer (2 μL) was added, loaded onto a 2% agarose gel and run at 100V for 30 min.

### Statistical analysis

Statistical significance was determined with paired Student’s t-test in SPSS Statistics v.22. P < 0.05 was considered statistically significant. All data are expressed as the means ± SEM.

## Results

### Primary culture of mouse SG epithelial cells

**Fig 1:**
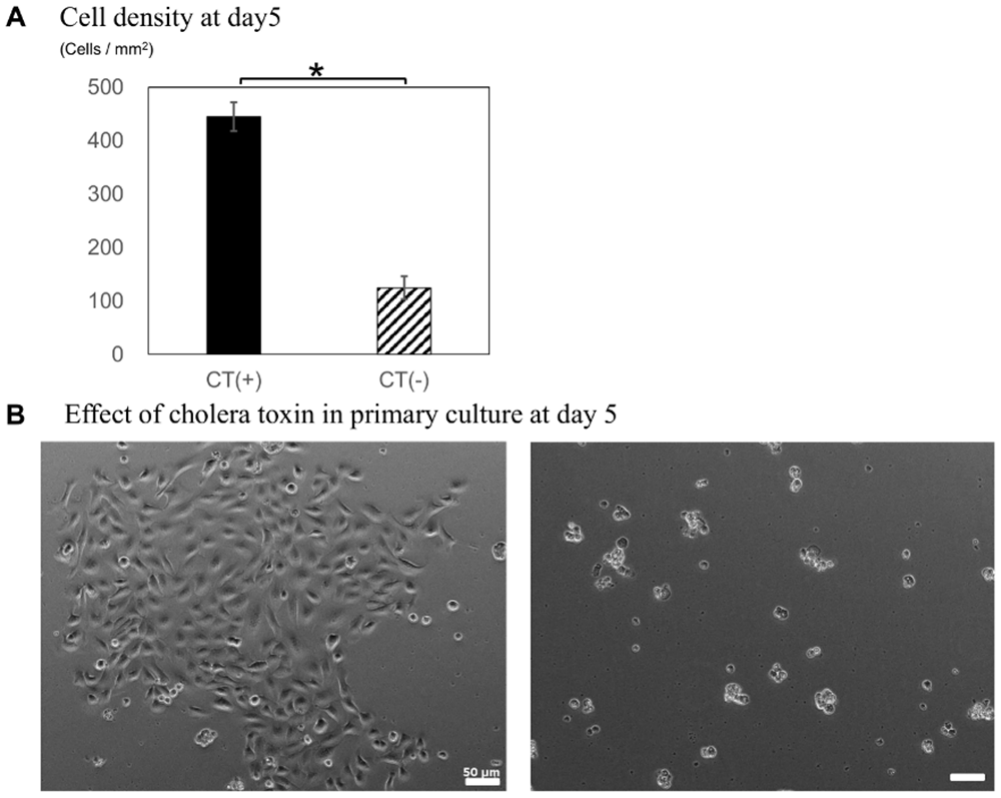
Effect of cholera toxin (CT) in primary mouse SG epithelial cell cultures. (A) Effect of CT on SG cell proliferation at day 5. (B) Morphology of the cells described in B. *p < 0.05, Scale bars = 50 μm.

Murine SGs were excised from the anterior neck between the submandibular lymph nodes and sternum. Hematoxylin and eosin (H-E) staining revealed comparatively more mucosal glands than serous glands; occasional duct structures and several acinar structures were also observed. Dissociated SG cells were cultured in low calcium, serum-free medium supplemented with epithelial growth factor (EGF) and CT, which are known to inhibit fibroblast growth and accelerate epithelial cell growth, respectively. Notably, the sparse epithelial cell cultures observed on day 1 developed into large colonies after 5 days in the presence of CT (*p* = 0.00011; Fig 1A), whereas control cultures failed to propagate and displayed marked cell death (Fig 1B). These data indicated that CT facilitated the selective culture of epithelial cells.

### Colony formation of mouse SG epithelial cells

**Fig 2:**
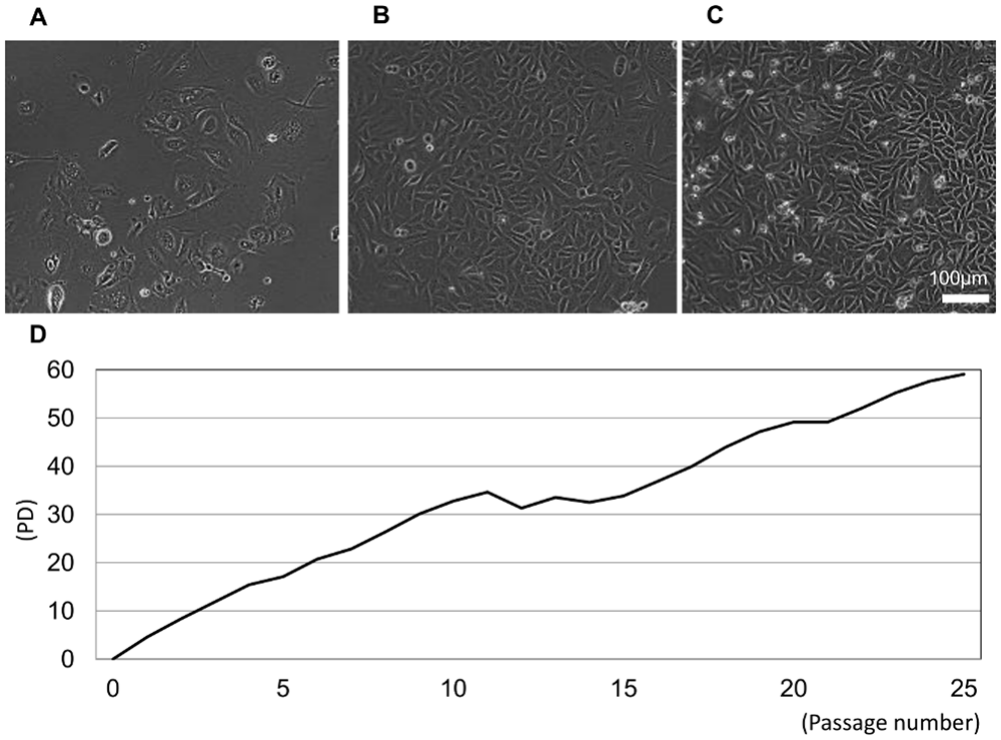
Subculture of mouse SG epithelial cells. Semiconfluent cell cultures at P1 (A), P10 (B), and P20 (C) at day 7 are shown. (D) Population doubling (PD) time of expanded SG epithelial cells over 25 passages. Scale bar = 100 μm.

Semiconfluent primary cell cultures displayed polygonal or fusiform morphology that gradually progressed into uniform cobblestones (Fig 2A–2C). The PD time of cultures over 25 passages is summarized in Fig 2D. This analysis revealed that epithelial SG cells exhibited stable proliferation, which stalled briefly during P10–P15, and recovered thereafter. Slower growth was observed at ~P20, which was ameliorated by increasing the frequency of medium changes to three times a week and using medium conditioned with supernatant from subcultured passages.

### Limiting dilution colony formation assay

**Fig 3:**
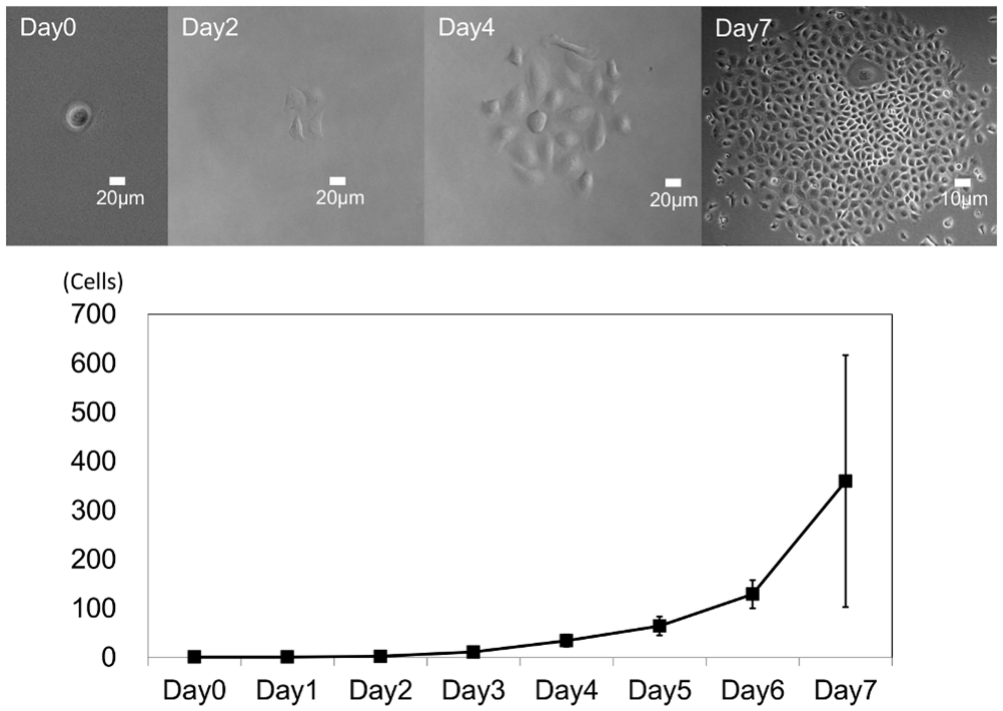
Clonogenic growth analysis by limiting dilution culture. Quantification of cell proliferation by clonogenic assay. Day 0–4 scale bars = 20 μm (×200); Day 7 scale bar = 10 μm (×100).

SG cells reached their Hayflick limit around P20-25; thus, limiting dilution assays were used to obtain cell colonies with higher proliferative potentials. Cultures were passaged once a week until 80% confluency, with medium changes twice per week. Single cells were seeded in 96-well plates and attached to the plate within 2 or 3 h. In turn, multiple colonies with similar morphology developed by day 7 (Fig 3).

### Long-term cultured SG epithelial cells

**Fig 4:**
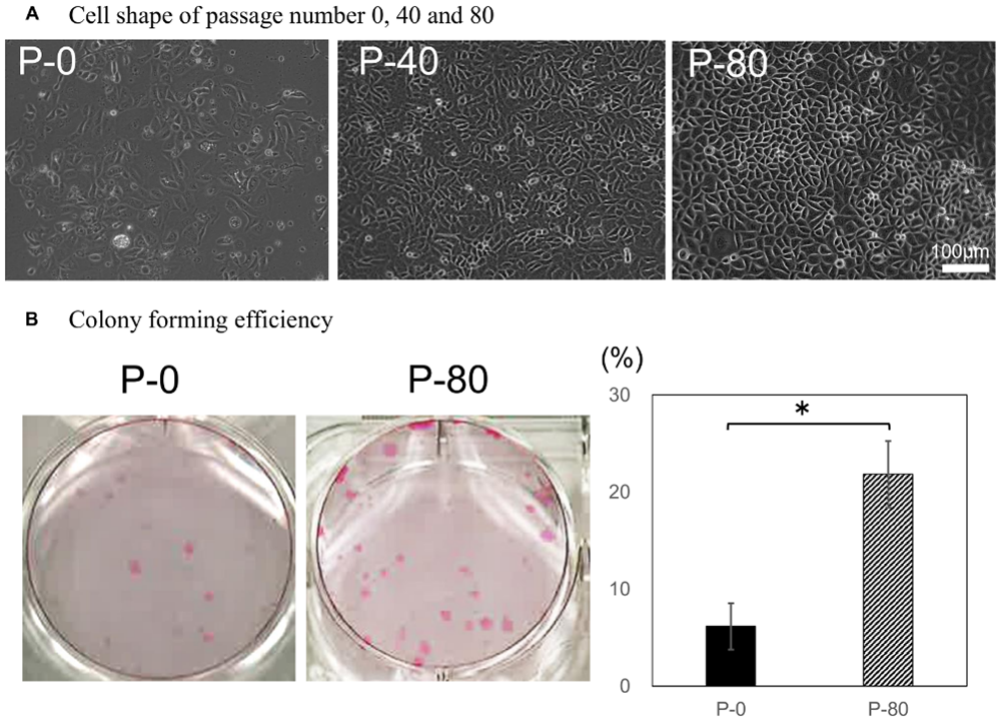
Long-term culture of SG epithelial cells derived from a single cell. (A) Cell morphology was monitored at P0, P40, and P80. Scale bar = 100 μm. (B) Colony formation was examined in P0 and P80 cells (n = 6). *p < 0.05.

Single-cell cultures (P25) were maintained for over 80 passages. Although primary (P0) cultures displayed mixed phenotypes with both elongated and cobblestone cells, the phenotypes of P40 and P80 cultures exhibited a similar cobblestone appearance (Fig 4A). Analysis of P0 and P80 cells revealed that P80 cells had a significantly higher colony forming efficiency (46.3% ± 19.3%) with colonies 1–2 mm in diameter, whereas no colonies were found in P0 cells (*p* = 0.0001; Fig 4B).

### Immunohistochemistry of mouse SG epithelial cells

**Fig 5:**
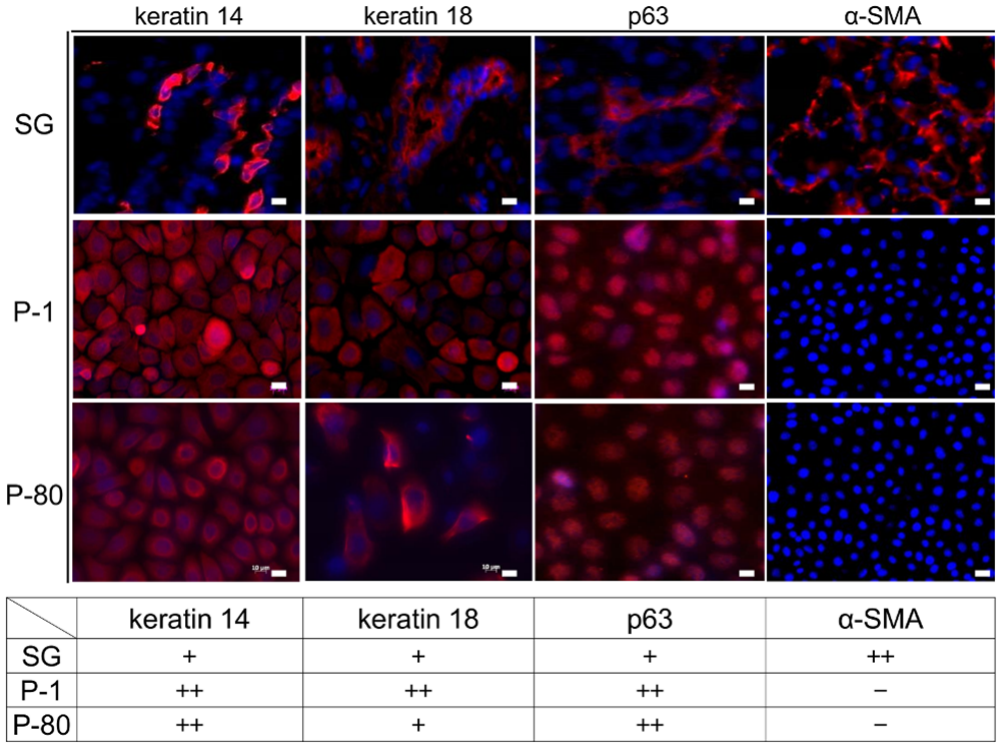
Immunohistochemistry of mouse SG epithelial cells. Immunohistochemical analysis of keratin 14, keratin 18, p63, and alpha-smooth muscle actin (α-SMA) expression in normal SG tissue (SG) and P1 and P80 cell cultures. Scale bars = 10 μm.

Keratin 14 is an epithelial cell marker that is highly expressed in the basal layer, but is expressed at lower levels within the more apical layers. In SG, keratin 14 was expressed around the acinar unit and duct structure. Immunohistochemical analysis of normal SG tissue and P1 and P80 cultures revealed that keratin 18 localized to the ducts of SG tissue and was expressed at higher levels in P1 cells than that of P80 counterparts (Fig 5). Moreover, expression of the basal epithelial cell marker p63 was found within the nuclei of SG tissue and cultured cells, while the myoepithelial cell marker α-SMA was expressed around acinar units in normal tissue, but decreased notably with subculturing. Collectively, long-term cultured cells expressed keratin 14, keratin 18, and p63 at levels phenotypically similar to those noted for basal cells of the ductal epithelium. These expression levels were recorded and shown in the below table. The staining levels were classified according to the relative percentage of the field of view when using the 40x objective (400x total magnification). 0% was designated as (−), 1–50% (+) and 51–100% (++).

### Comparison of gene expression in SG tissue and cell cultures

**Fig 6:**
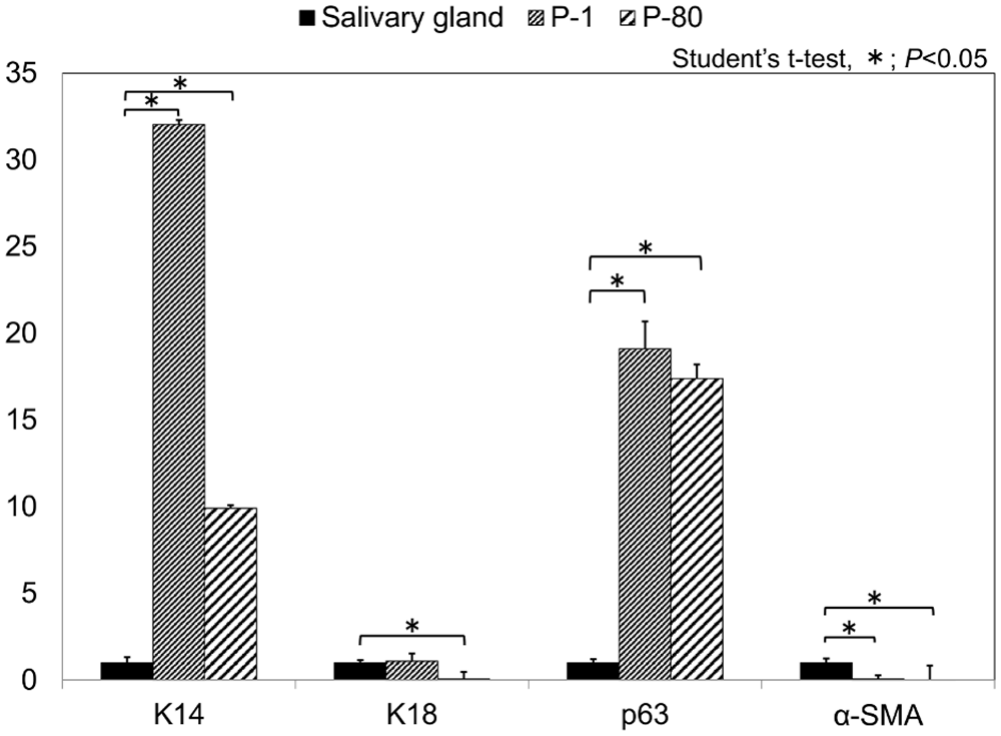
Quantitative real-time PCR analysis of gene expression in SG tissue and P1 and P80 cell cultures. Keratin 14, keratin 18, p63, and α-SMA gene expression were analyzed in SG tissues and P1 and P80 cell cultures by qRT-PCR analysis. *p < 0.05.

Gene expression analysis of keratin 14, keratin 18, α-SMA, and p63 in SG tissue and P0 and P80 cultures were monitored by quantitative real-time PCR (qRT-PCR). Notably, keratin 14 and p63 gene expression were significantly lower in SG tissue (keratin 14 in P0 and P80 vs. SG, *p* = 0.00023 and 0.0000079, respectively; p63, *p* = 0.017 and 0.0000543, respectively). Interestingly, α-SMA and keratin 18 expression decreased with prolonged culture (α-SMA, both *p* < 0.001; keratin 18, *p* = 0.219 and < 0.001, respectively). This result demonstrated that P80 cells retain a phenotype similar to that of early ductal epithelial cell cultures, but would likely progress to a more basal epithelial phenotype with longer culture periods. (Fig 6)

### Effect of CT on colony formation, adherence, and cell proliferation of primary and late cultures

**Fig 7:**
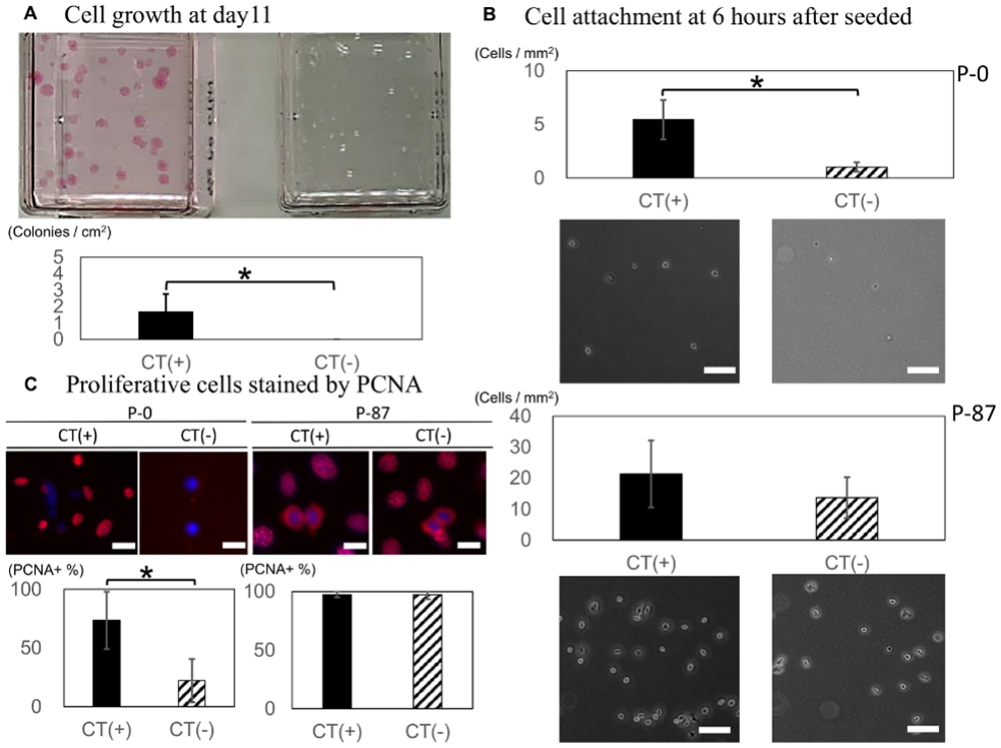
CT enhanced cell attachment and proliferation specifically in primary cells. The effect of CT on (A) proliferation, (B) adherence, and (C) PCNA expression in primary (P0) and long-term (P87) cell cultures. *p < 0.05, Scale bars = 10 μm.

Analysis of CT-mediated effects on colony formation after 11 days in culture showed that significantly greater number of colonies formed from P87 cultures than for the P0 counterparts (*p* = 0.0068; Fig 7A). In addition, plated cells displayed a greater potential for adherence when cultured in the presence of CT (*p* = 0.00000076; Fig 7B); however, this difference trended toward significance only in P87 cultures (*p* = 0.072). Comparison of the adhesion rates of primary culture (P0) and late passage culture (P87) in the absence of CT showed a significant increase independent of CT (*p* = 0.00145). These findings were confirmed by PCNA immunohistochemistry for PCNA expression (Fig 7C), which showed no significant differences with respect to CT in P87 cultures (*p* = 0.873). However, P0 cells cultured in the presence of CT displayed significantly higher expression of PCNA than control cultures (*p* = 0.029).

### Mouse cell line authentication

Gene amplification from human STR markers were absent in our cell line. Furthermore gene amplifications from mouse STR markers, 4–2, 6–7, 15–3 and 6–4 were observed. Therefore cell line we established was animal origin and validation of the mouse strain was obtained.

## Discussion

In this study, we found that SG epithelial cells derived from wild type mice could be cultured for over 80 passages while maintaining their phenotype and proliferative ability. Our results emphasize the importance of culturing in low calcium, serum-free medium, as well as the addition of CT during the first several passages in order to isolate epithelial cells. CT supplementation was used for 10 passages because cell growth was not stable; however, results from PCNA staining suggested that this period using could be shorter than this. Several salivary gland cell lines have been established following retroviral transduction of SV40 large T antigen.[19] Laoide et al. established the immortalized mouse submandibular epithelial cell lines SIMS and SIMP from 12-day-old and 22-day-old mice transgenic for SV40 large T antigen or polyoma viruses, but these were non-transformed ductal cell lines.[20] Modified culture conditions have been investigated to overcome such immortalization procedures. For instance, Hiraki et al. reported that calcium induces the formation of well-developed cytoplasmic organelles and increases alpha-amylase expression in primary human salivary acinar cells,[21] whereas Dimitriou et al. demonstrated that serum-free culture medium supplemented with essential epithelial growth factors was sufficient to maintain human non-neoplastic salivary gland epithelial cells in vitro for prolonged time periods.[22] Others have used combinations of primary explant culture and insulin and hydrocortisone to enhance proliferation.[23] Interestingly, numerous studies have demonstrated that CT enhanced primary epithelial culture viability by facilitating cell proliferation and adherence. In addition, Greene et al. established epidermal keratinocyte line using CT-supplemented nutrient medium and suggested that CT supported epidermal cornification and suppressed fibroblast growth.[24] Ma et al. reported that long term culture and growth kinetics of murine corneal epithelial cells expanded from single corneas by serum-free, low-calcium medium containing CT [25]. We reported on the generation of stratified epithelial sheets engineered from a single adult murine corneal/limbal progenitor cell using similar conditions. [23–26] Thus, cultures in the present analyses were maintained in standard epithelial cell culture media containing CT, resulting in an emergent population of epithelial cells at day 5 (*p* = 0.00011). Additionally, these cells maintained in an undifferentiated state with no marked changes in morphology, suggesting that these culture conditions might be necessary to culture wild type murine SG epithelial cells.

Currently available salivary gland epithelial cell lines can be classified into acinar-like or ductal-like phenotypes. HSG and HSY are popular tumor-derived cell lines with morphological characteristics similar to those of intercalated duct cells that secrete amylase and differentiate into acinar structures when cultured in Matrigel^®^[27, 28], both of which are well established in vitro models for salivary gland secretion and morphology. It is exceedingly difficult for cells to retain an acinar structure in 2D in vitro culture systems and must be tumor-derived or immortalized with exogenous factors to maintain proliferation ability. However, cells 2D-cultured cells immediately differentiated into epithelial morphology and failed to maintain an acinar or spherical structure; thus, basal duct cells can more easily be cultured in vitro than acinar or myoepithelial cells, but are difficult to establish from wild type while also retaining a long-term phenotype. In this study, we overcame this hurdle using CT and a cell matrix substrate to generate cell lines with stable proliferation. While CT is believed to accelerate cell growth by elevating intercellular cyclic AMP concentrations [24, 29]; it is more difficult to determine the direct effect of cell adherence on viability. We suggested that suitable adherence was derived from acquisition of proliferation and cell adhesive substrate such as gelatin because substrate coated with gelatin promote monolayer growth.[30] Phenotypic analysis of primary and long-term SG epithelial cell cultures were examined by immunohistochemistry and qRT-PCR analyses for keratin 14, keratin 18, and p63, which reflected basal [31, 32], ductal [33, 34], and basal duct cell [35, 36], respectively. These experiments revealed that cultured cells were phenotypically similar to basal cells of the ductal salivary gland epithelium. Additionally, in passages 20, 50 and 80, cells did not form aggregates and salivary spheres in suspension culture; thus, obvious cellular transformation was not observed. Our cell line also exhibited a ductal phenotype, and thus might be capable of forming acinar or myoepithelial cells. Thus, cell differentiation and secretory functions should be evaluated in future analyses. In this research, mouse cell line authentication was performed by STR analysis. But because several gene amplifications from mouse STR marker were absent, additional exploration was needed. Furthermore cells appeared to go through a crisis stage seen as a pause in doubling about passage 11 in Fig 2, karyotype analysis is an important subject of future investigation and should be left open.

In conclusion, SG epithelial cells derived from wild type mice were successfully cultured for 80 passages in low calcium, serum-free media supplemented with CT. The established cell line was phenotypically similar to basal cells of the salivary gland ductal epithelium in vivo and may be a beneficial cell model for studying salivary gland regeneration in the future.

## Supporting Information

S1 Fig Gene amplification from mouse and human STR markers. Lanes labeled A on the gel contained the DNA ladder (100–1500 bp) and lane M contained the β-actin. Lanes B—J were mouse STR markers and K, L were human. B = 18–3; C = 4–2; D = 6–7; E = 9–2; F = 15–3; G = 6–4; H = 12–1; I = 5–5; J = X-1; K = D8S1106; L = D4S2408.
(TIF)
