# Supplementary figures and images for: Characterization of Long-Term Cultured Murine Submandibular Gland Epithelial Cells
Source: PLoS One. 2016 Jan 22;11(1):e0147407. doi: 10.1371/journal.pone.0147407 (PMC4723076; doi:10.1371/journal.pone.0147407)

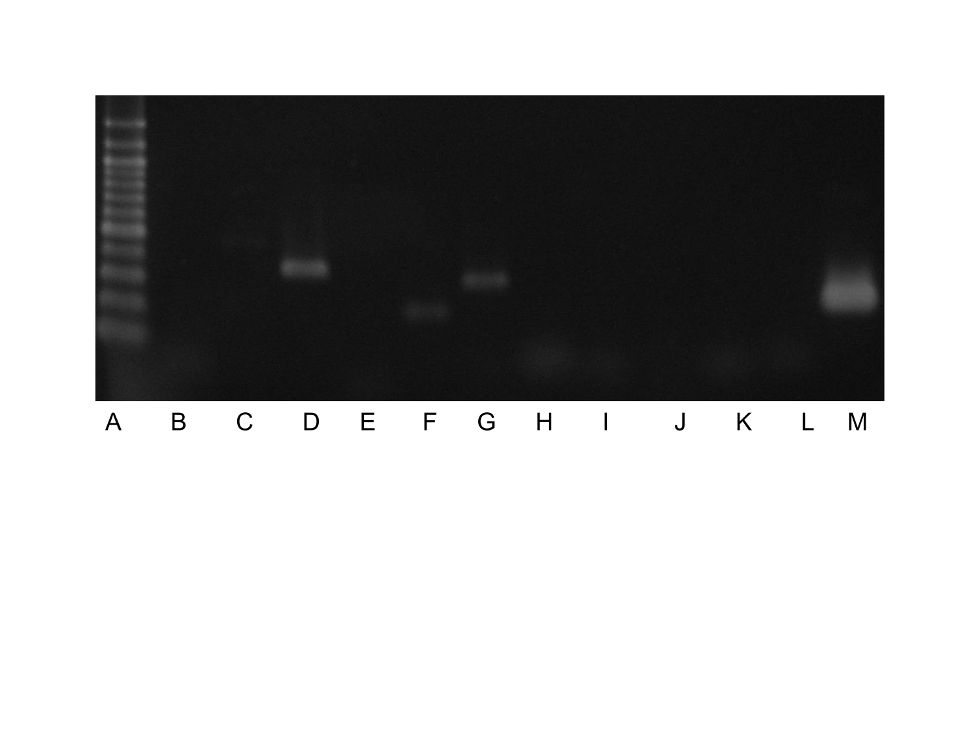

Supplement: S1 Fig — Lanes labeled A on the gel contained the DNA ladder (100–1500 bp) and lane M contained the β-actin. Lanes B—J were mouse STR markers and K, L were human. B = 18–3; C = 4–2; D = 6–7; E = 9–2; F = 15–3; G = 6–4; H = 12–1; I = 5–5; J = X-1; K = D8S1106; L = D4S2408. (TIF) [file pone.0147407.s001.tif]
